# Radiomics Analysis of Contrast-Enhanced CT for Hepatocellular Carcinoma Grading

**DOI:** 10.3389/fonc.2021.660509

**Published:** 2021-06-04

**Authors:** Wen Chen, Tao Zhang, Lin Xu, Liang Zhao, Huan Liu, Liang Rui Gu, Dai Zhong Wang, Ming Zhang

**Affiliations:** ^1^ Department of Medical Imaging, The First Affiliated Hospital of Xi’an Jiaotong University, Xi’an, China; ^2^ Department of Radiology, Taihe Hospital, Hubei University of Medicine, Shiyan, China; ^3^ Precision Medicine Research Center, Taihe Hospital, Hubei University of Medicine, Shiyan, China; ^4^ GE Healthcare, Shanghai, China; ^5^ Department of Radiology, Shanghai Sixth People’s Hospital, Shanghai, China; ^6^ Department of Pathology, Taihe Hospital, Hubei University of Medicine, Shiyan, China

**Keywords:** radiomics, machine learning, support vector machine, hepatocellular carcinoma, grading

## Abstract

**Objectives:**

To investigate the value of contrast-enhanced computer tomography (CT)-based on radiomics in discriminating high-grade and low-grade hepatocellular carcinoma (HCC) before surgery.

**Methods:**

The retrospective study including 161 consecutive subjects with HCC which was approved by the institutional review board, and the patients were divided into a training group (n = 112) and test group (n = 49) from January 2013 to January 2018. The least absolute shrinkage and selection operator (LASSO) was used to select the most valuable features to build a support vector machine (SVM) model. The performance of the predictive model was evaluated using the area under the curve (AUC), accuracy, sensitivity, and specificity.

**Results:**

The SVM model showed an acceptable ability to differentiate high-grade from low-grade HCC, with an AUC of 0.904 in the training dataset and 0.937 in the test dataset, accuracy (92.2% versus 95.7%), sensitivity(82.5% versus 88.0%), and specificity (92.7% versus 95.8%), respectively.

**Conclusion:**

The machine learning-based radiomics reflects a better evaluating performance in differentiating HCC between low-grade and high-grade, which may contribute to personalized treatment.

## Introduction

Hepatocellular carcinoma (HCC) is the most common malignant tumor and predicted to the fourth leading cause of cancer death worldwide in 2018 (1–3). More than 300,000 people died in China because of liver cancer every year, accounting for 51% of liver cancer deaths worldwide (3). Surgical resection is the most effective treatment for patients with HCC. Patients who meet Milan criteria or undergo down-staging of their tumors to be within the Milan criteria are preferred for live transplantation (4, 5). The survival rates exceed 70% during 5 years, with recurrence in less than 15% of patients, who met Milan criteria and received a liver transplant (6). HCC is prone to metastasis and recurrence. However, the long-term prognosis of HCC patients is still unsatisfactory, although favorable results in terms of survival and recurrence have been reported based on highly selected patients (7, 8). The pathological grading of HCC plays an essential role in determining the patient’s prognosis. The current study reflected that pathological grading is a risk factor of overall early recurrence in HCC (9, 10). Intrahepatic recurrence and extrahepatic metastasis are more likely to occur in high-grade HCC tumors than low-grade tumors (11). Therefore, accurate preoperative prediction for HCC grading is crucial for treatment planning.

Medical imaging patterns of non-invasive contrast-enhanced CT in HCC patients are essential for accurate estimates of the clinical-stage, prognosis clinical decision-making, and determination of follow-up in primary hospitals. Contrast-enhanced CT provides information about tumor vascularization. Studies have shown that there was a significant correlation between pathological grade and radiological enhancement on contrast-enhanced CT (12), Only 46 patients were included in the study. Texture analysis based on CT images was reported to evaluate the differentiation and grade both of pancreatic carcinoma esophageal and renal cell carcinoma (13, 14).

Radiomics converts imaging data into a high-dimensional mineable feature space using many automatically extracted data-characterization algorithms (15, 16). Quantitative features based on intensity, shape, and texture could be reflected in much information on tumor phenotype (17). Radiomics signatures have been proven to reflect tissue heterogeneity (18, 19). CT-based radiomics has been proven to discriminate tumor stage and grade in colorectal cancer (20, 21). Recent studies have shown that there is a correlation between medical imaging based on texture features and radiomics signatures and pathological grading (22–24).

However, there are few studies based on thin-portal phase CT radiomics to predict hepatocellular carcinoma grade. Therefore, we aimed to investigate the value of contrast-enhanced CT-based on portal venous radiomics signatures to distinguish HCC grade preoperatively.

## Materials and Methods

### Patients

**Figure 1:**
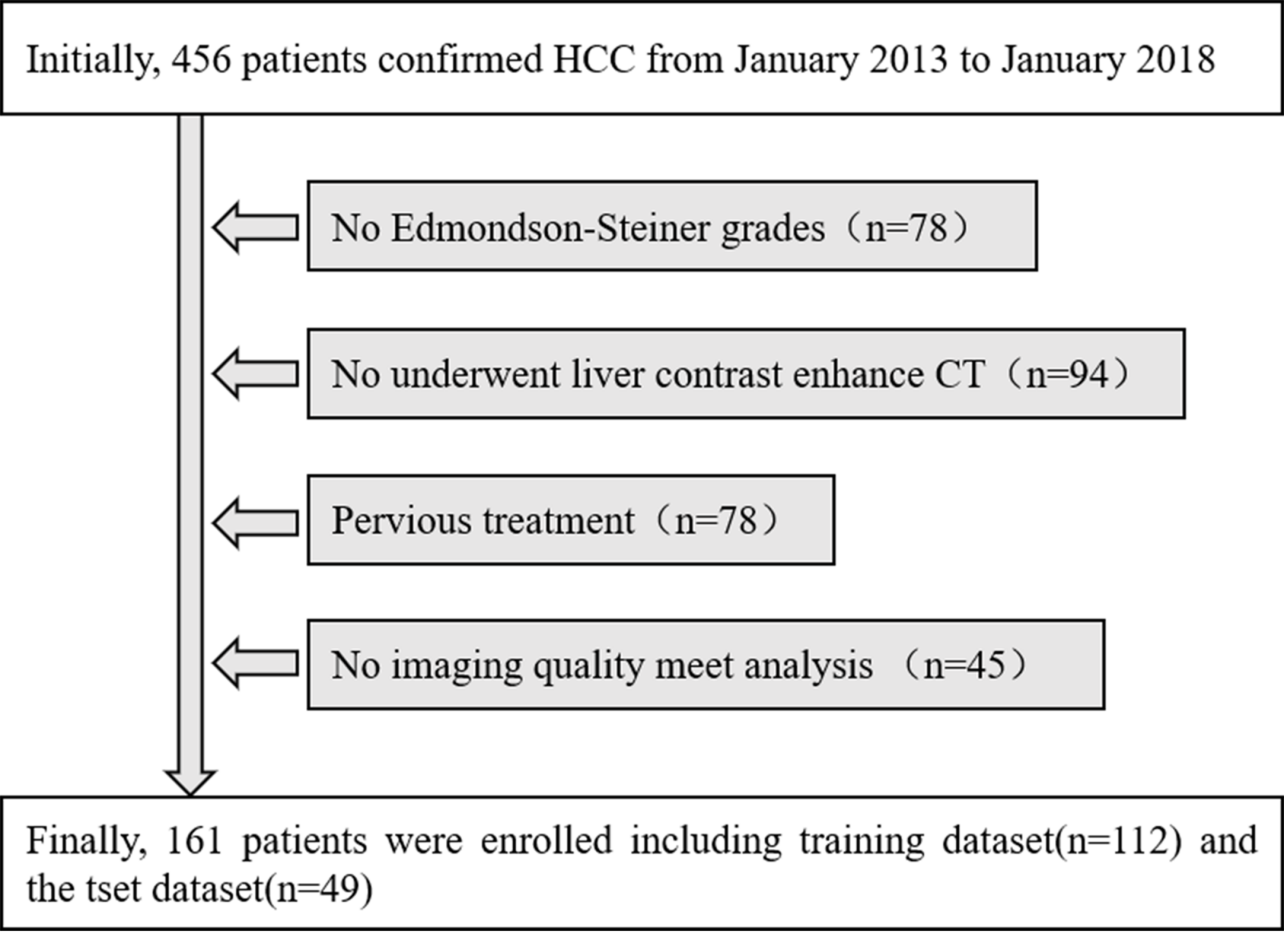
Flowchart of the inclusion and exclusion processes.

The retrospective study was approved by the institutional review board, and the informed consent requirement was waived. The inclusion criteria were as follows: (1) patients who underwent surgical resection and were pathologically confirmed as HCC with a usable histological report according to Edmondson–Steiner grades; (2) patients who underwent liver contrast-enhanced CT within two weeks before operation; (3) Without previous treatment with patients such as radiofrequency ablation, transcatheter arterial chemoembolization (TACE), liver resection or percutaneous ethanol injection; and (4) the quality of the images satisfied the needs of analysis and have completed the portal venous phase CT images and clinical and pathological data. The patients with HCC with a contrast-enhanced CT examination in our institute were recruited from January 2013 to January 2018. The enrolled 161 patients with HCC, were classified into the training dataset [112 patients; 86 males (76.8%) and 26 women (23.2%)], with a median age of 53 years (range 25 to 71 years)] and the test dataset [49 patients; 40 males (81.7%) and nine females (8.3%), with a median age of 57 years (range 28 to 74 years)]. Baseline clinicopathologic data, including gender, age, preoperative AFP level, were derived from the institution archives. Finally, a total of 161 subjects were selected from the total 456 patients in our research, and the details are shown in **Figure 1**.

### Assessment of Histologic Grade

Histological grading data of HCC tumors were obtained from pathology reports reviewed by the pathologist. Histological grade was postoperatively determined as low- and high-grade. Edmondson grades I, I–II, and II correspond to Low-grade tumors, and Edmondson grades II–III, III, III–IV, and IV correspond to high-grade tumors (25). There was inconsistent differentiation in the tumor. Tumor cells of different pathological grades could be contained in the same mass. The larger one is determined as the pathological grade of the tumor (26).

### Image Acquisition

All patients underwent 64 slices multidetector CT scanner of the liver (Optima CT660 or LightSpeed VCT, GE Healthcare), parameters were as follows: for non-enhanced studies and the hepatic arteriovenous phase, the gantry rotation time is 0.6 s, and the equilibrium phase is 0.8 s; the cross-sectional thickness is 5 mm; the table speed is 27.5 mm/s; 120 kVp; and 160–440 mA. Patients imaged with a CT scanner in a craniocaudal direction. The scan range is from the dome to the lower liver. Non-ionic contrast medium (Iohexol Injection) administered at a total dose of 70–80 ml based on body weight (0.9 ml/kg), 2.5–3.0 ml/s through a 20 gauge venous cannula placed in the antecubital vein. For triphasic acquisitions, scanning started with a 30 s scan delay (about 30–35 s after injection of the contrast agent) for the hepatic arterial phase. Thirty-five seconds after the endpoint of the hepatic arterial phase (about 65–70 s after injection of the contrast agent), the scans for the portal venous phase were obtained. Delayed phase images reached 120 s (about 150–180 s after injection of the contrast agent) (27).

### Tumor Segmentation

**Figure 2:**
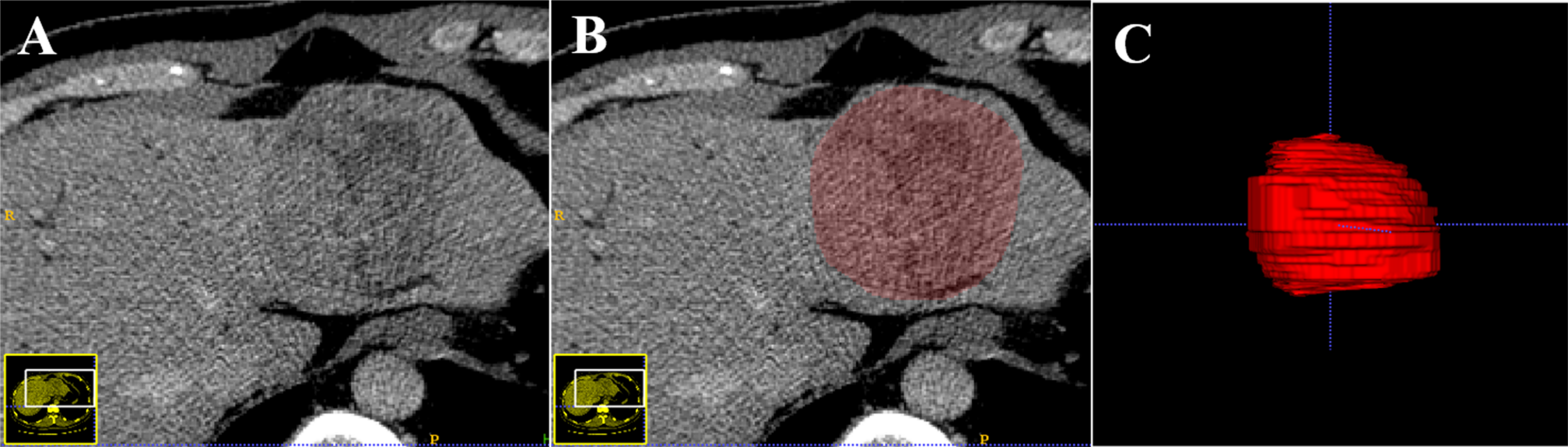
An example of the manual segmentation in hepatocellular carcinoma. The portal venous phase computed tomography (CT) image **(A)**. Manual segmentation on the same axial slice **(B)**. Generation of a 3D ROI **(C)**.

Tumor segmentation was performed on the portal venous phase CT images, retrieved from the picture archiving and communication system (PACS). The images were loaded into the ITK-SNAP software (open-source software http://www.itksnap.org) for manual segmentation, and a three-dimensional volume of interest (VOI) that covered the whole tumor was delineated in the images respectively segmented by a radiologist with over five years of experience in abdominal imaging. The procedure is shown in **Figure 2**.

### Radiomics Feature Extraction and Selection

After integrating the VOI that covered the whole tumor images, a three-dimensional radiomics feature was extracted from the CT images with the Artificial Intelligence Kit software (AK, version 3.2.2, GE Healthcare). A total of 396 radiomics features from each patient were generated based on the following five categories: Histogram, shape, Gray-level co-occurrence matrix (GLCM), Gray-level size zone matrix (GLSZM), and Run-length matrix (GLRLM). Most features defined comply with feature definitions as described by the Imaging Biomarker Standardization Initiative (IBSI).

Thirty CT images were randomly chosen for the second segmentation by two experienced radiologists (twice by reader one and once by reader 2, with eight and thirteen years of clinical experience in the abdominal study). Intra- and interclass correlation coefficients (ICC) were applied to assess the stability and reproducibility to find out robust features. Based on the twice feature extraction by reader 1, the intra-observer ICCs were calculated. Meanwhile, the interobserver ICCs were obtained based on the first-extracted features by reader one and those by reader 2. Generally, ICC >0.75 was considered to be excellent in reproducibility (28). The remaining tumor segmentation for feature extraction was performed by reader 1.

**Figure 3:**
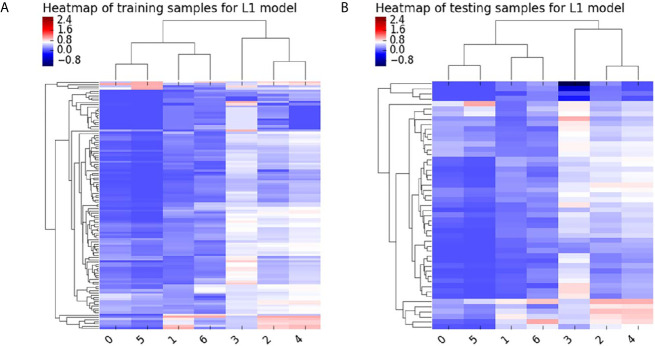
Heatmap of the model in the training **(A)** and test samples **(B)** for L1 model.

All patients were randomly divided into two independent datasets with a ratio of 7:3 using stratified sampling. The feature scaling method was employed before dimensionality reduction to decrease the difference in radiomics features. First, The general univariate analysis was used to select features. The least absolute shrinkage and selection operator (LASSO) was applied to select the most useful features from the primary data in the training dataset. The Heatmap of the model in the training and test samples is shown in **Figure 3**. Detailed radiomics parameters and remained features are shown in the **Supplementary Material**.

### Establishment of the Model Based on Machine Learning

The most predictive features were applied to establish an optimal SVM model using a grid search method with 5-fold cross-validation. The AUC, sensitivity, specificity, positive predictive value (PPV), negative predictive value (NPV), and accuracy were further calculated in the dataset. And independently validated in the test dataset to evaluate prediction accuracy.

### Statistical Analysis

Continuous variables were analyzed with the Shapiro–Wilk test to determine their distributions. The Student’s t-test and the Mann–Whitney U test were used to determine whether the characteristic features were significantly different between the low- and high-grade HCC groups in the training dataset and test dataset. The statistical significance levels reported in this study were all two-sided, and a *P*-value <.05 was considered statistical significance. All statistical analyses were performed using IPMs 2.0 (IPM statistics, GE healthcare).

## Results

### Clinical Characteristics and Pathologic Findings

The training dataset consisted of 86 males and 26 females. The mean age in the low grade of HCC in the training dataset is 56.45 ± 10.44; range from 20–69 years. The mean age of high grade is 49.74 ± 8.58 years; content from 25–78years, which has a significant difference (*p* <0.01). The test dataset included 40 males and nine females (mean age, 51.38 ± 8.22; range, 24–83 years in low grade; mean age, 51.88 ± 10.74; range, 25–72 years in high grade). No statistically significant differences existed in gender between the training and the test datasets (*p* = 0.317; *p* = 0.662). Clinical characteristics were detailedly shown in **Table 1**. No significant difference was found in the AFP level between patients with low-grade and high-grade HCC either in the training dataset or test dataset (*p* = 0.186; *p* = 0.150). Among 161 patients underwent surgical resection, including laparoscopy or laparotomy. Of these, 79 were low-grade hepatocellular carcinoma, and 82 were high-grade hepatocellular carcinoma.

**Table 1:** Baseline characteristics of patients in training dataset and test dataset.

Characteristics	Training dataset			Test dataset		
	Low Grade	High Grade	*P* value	Low Grade	High Grade	*P* value
Age, mean ± SD, y	56.45 ± 10.44	49.74 ± 8.58	<0.01	51.38 ± 8.22	51.88 ± 10.74	0.855
Gender<N						
Male	40	46	0.317	19	21	0.662
Female	15	11		5	4	
AFP (ug/L) Median	20.5	32.4	0.186	20.55	27.3	0.15
(IQR)	(8.21, 42.3)	(7.82,45.95)		(6.95,35.54)	(10.54,61.82)	

### Reproducibility of Radiomics Feature Extraction

**Figure 4:**
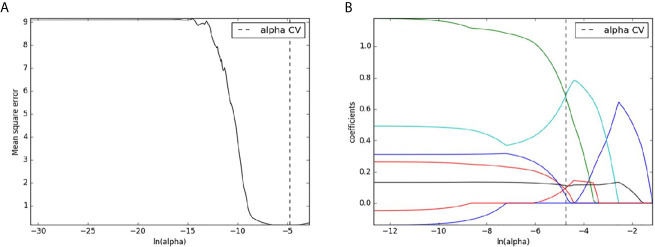
Radiomics features selection with LASSO binary logistic regression method. The mean square error was plotted versus the In (alpha) sequence **(A)**; The coefficient profile plot was plotted versus the In (alpha) sequence **(B)**.

A total of 396 radiomics features were extracted for each patient. Among these radiomics features, 312 features were considered excellent reproducibility with ICC >0.75 in intra-and interobserver. Therefore, the 312 robust features of each patient were used for further selection. Finally, seven features with non-zero coefficients were eventually remained from the 312 radiomics features using LASSO logistic regression (**Figure 4**).

### Performance of SVM

**Figure 5:**
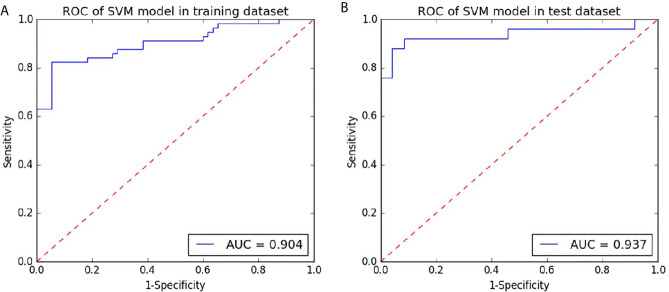
Receiver operating characteristic curves (ROC) of the portal phase CT-based SVM for preoperative prediction of the grade of hepatocellular carcinoma in the training and testing datasets. **(A)** the ROC curve of the radiomics signature based on the portal phase CT based on the training dataset. **(B)** the ROC curve of radiomics signature based on the portal phase CT for the test dataset.

The model based on SVM on the portal venous phase CT images performed well on high-grade patients from low-grade patients. With an AUC of 0.904 in the training dataset, the test dataset with an AUC of 0.937 (**Figure 5**). The other predictive parameters (sensitivity, specificity, PPV, NPV, and accuracy) of SVM are shown in **Table 2**.

**Table 2:** The predictive performance of the SVM model for preoperative the grade of hepatocellular carcinoma based on contrast-enhanced CT.

Predictive Performance	AUC	SEN	SPE	ACC	PPV	VPV
Training dataset	0.904	0.825	0.927	0.922	0.922	0.836
Test dataset	0.937	0.880	0.958	0.957	0.957	0.885

## Discussion

Hepatocellular carcinoma (HCC) is the most common tumor of liver cancers accounting for more than 90% (6). Most of the world’s HCC cases are found in the Asia-Pacific region, where annual HCC-related mortality rates have risen significantly over the last 20 years (29). HCC has become a major emerging public health problem in the Asia-Pacific region. Radiomics has been proven useful in tumor grade in clear cell renal cell carcinoma, soft tissue sarcomas and Colorectal Adenocarcinoma (21, 30, 31). The SVM classifier, a specific type of supervised machine-learning method, has been used to predict the grade of the glioma (32–34) and clear cell renal cell (35). As the results showed, The SVM model was finally developed after the LASSO regression analysis to discriminate the grade of HCC in both the training dataset (*p <*0.05) and the test dataset (*p <*0.05). It indicates that radiomics features on the portal venous phase CT images can be used to detect tiny differences in the density of tumors.

The AUC on the portal phase CT-based SVM for preoperative prediction of the grade of hepatocellular carcinoma is 0.904 and 0.937 in the training and test datasets. A published study showed that the radiomics signature based on T1WI or T2WI images showed performance in predicting the HCC grade (with AUC of 0.712 and 0.722 in the test dataset) (22). Mao et al. showed that the radiomics signatures based on arterial phase Contrast-enhanced computed tomography images could successfully distinguish pathological grades of HCC, with the AUC of 0.731 and 0.718 in the training and test datasets (24), which are lower than the AUC of the SVM model developed in our study either in training dataset or test dataset. This result may be related to section thickness and selection of the various phases of contrast enhancement. The section thickness chose is 0.625 or 1.25 mm and we chose the portal venous phase in our study. Approximately 80–90% of HCCs are hypervascular lesions, arterial phase of imaging could increase enhancement in the tumor parenchyma. Due to differences in tumor biology, measurements of HCC will vary at the various phases of contrast enhancement (36). Mapping the size of hepatocellular carcinoma (HCC) on images plays an important role in accurately capturing a three-dimensional region of interest. Research has suggested that the portal venous phase may be optimal for measuring HCC on MRI (37), which is similar to CT. They hypothesized that benign reactivity or perfusion-related changes may occur in the liver parenchyma around the tumor during the arterial phase and may present as transient congestion during this phase, leading to measurement bias. The tumor will be with wash out on the portal venous phase, making it easier to show the boundary of the tumor.

Studies have shown that the texture features based on arterial phase CT images are associated with pathological grades of HCC (23). Texture feature based on Gd-DTPA-enhanced MR images showed better diagnostic efficacy (with AUCs of 0.827–0.918) (38) because MR images providing more information about tumor heterogeneity. However, the case number was limited, It may lead to a possible risk of data overfitting.

Some studies indicate that the model which combined clinical factors with the radiomic model outperformed compared with other models (22, 24). Research shows AFP level was an independent factor that could discriminate between high-grade and low-grade HCC (22). However, there is no significant difference between high-grade and low-grade HCC in both the training and test datasets (*p* = 0.186 and *p* = 0.15). It may be due to the high AFP level in some patients in this study, resulting in the imbalance of the AFP level.

There are several limitations to our research. First, the number of HCC was limited and it was a single-center retrospective study. Therefore, more cases are needed for future studies and further multicenter cohorts should be conducted. Second, the portal venous phase image was merely considered, which might somehow miss some useful information for the hepatic arterial and hepatic venous phases. Thus, the phase images should also be incorporated in future studies. Third, the etiology of liver cancer hasn’t been classified. thus, further research is needed to determine whether our findings would be influenced by different etiology such as hepatitis B, hepatitis C-related liver diseases, and alcohol-related cirrhosis.

## Conclusions

An SVM model by radiomics signature based on contrast-enhanced CT images may be useful as a new approach to predicting the histological grade of HCC before the operation.

## Data Availability Statement

The original contributions presented in the study are included in the article/**Supplementary Material**. Further inquiries can be directed to the corresponding author.

## Ethics Statement

The studies involving human participants were reviewed and approved by Letter of Medical Ethics Committee of Shiyan Taihe Hospital. The patients/participants provided their written informed consent to participate in this study.

## Author Contributions

WC, TZ, and MZ reviewed and drafted the manuscript. TZ, RG, and DW did the literature search. LX and LZ modified the figures. HL and WC have revised and edited the manuscript. All authors contributed to the article and approved the submitted version.

## Funding

This study was funded by the research grant from the Health Commission of Hubei Province scientific research project (WJ2021M047).

## Conflict of Interest

Author HL was employed by company GE Healthcare.

The remaining authors declare that the research was conducted in the absence of any commercial or financial relationships that could be construed as a potential conflict of interest.
