# Supplementary material for: Radiomics Analysis of Contrast-Enhanced CT for Hepatocellular Carcinoma Grading
Source: Front Oncol. 2021 Jun 4;11:660509. doi: 10.3389/fonc.2021.660509 (PMC8212783; doi:10.3389/fonc.2021.660509)
Supplement: Supplementary file 1 [file DataSheet_1.docx]

**Texture analysis methodology**

Texture analysis wad applied to the CT images using in-house software (AK, version 3.2.2, GE Healthcare). A region of interest (ROI) was delineated initially around the tumor outline for the 3D ROI area. 396 imaging texture features from the category of histogram, the Grey level co-occurrence matrix(GLCM), the gray level size zone matrix(GLSZM), the gray level Run-length matrix(RLM), and Shape and size based features were finally extracted from one single image **Table S1.**

**Table S1:**

**Summary of radiomic features used in this study**

| Feature classes | No. of features | 3 representative features |
| --- | --- | --- |
| Histogram | 42 | FrequencySize, MaxIntensity, MeanValue,… |
| GLCM | 144 | ClusterProminence, ClusterShade, Correlation,… |
| GLSZM | 11 | SizeZoneVariability, HighIntensityEmphasis, IntensityVariability,… |
| RLM | 180 | GreyLevelNonuniformity, HighGreyLevelRunEmphasis, LongRunEmphasis,… |
| Formfactor | 9 | Compactness1, Maximum3DDiameter, Sphericity,… |
| Haralick | 10 | HaraEntroy, contrast, differenceEntropy,… |
| Total | 396 |  |

GLCM= the Grey level co-occurrence matrix, GLSZM =the gray level size zone matrix, RLM= the gray level Run-length matrix

312 features were considered excellent reproducibility with ICC > 0.75 in intra-and interobserver.

First, The general univariate analysis was used to select features.

parameters setted: {'P value for threshold in': 0.05}

num of remained features: 288

remained features:

[['ShortRunLowGreyLevelEmphasis_angle45_offset7']

['Correlation_angle90_offset1']

['ClusterShade_AllDirection_offset4']

['HaralickCorrelation_angle45_offset1']

['HighGreyLevelRunEmphasis_angle135_offset4']

['ClusterShade_angle90_offset4']

['HaralickCorrelation_angle90_offset1']

['ClusterProminence_angle0_offset7']

['Correlation_angle45_offset7']

['ShortRunEmphasis_AllDirection_offset1']

['Inertia_angle90_offset7']

['GreyLevelNonuniformity_angle135_offset7']

['InverseDifferenceMoment_angle135_offset4']

['Sphericity']

['Inertia_angle135_offset7']

['HaralickCorrelation_angle90_offset7']

['ClusterProminence_AllDirection_offset7_SD']

['GLCMEntropy_angle0_offset7']

['GLCMEnergy_AllDirection_offset4_SD']

['ShortRunEmphasis_angle135_offset7']

['ShortRunHighGreyLevelEmphasis_AllDirection_offset1_SD']

['Inertia_AllDirection_offset1']

['ShortRunEmphasis_angle45_offset1']

['GreyLevelNonuniformity_angle0_offset4']

['GLCMEntropy_angle0_offset1']

['Inertia_AllDirection_offset7_SD']

['GreyLevelNonuniformity_AllDirection_offset7']

['Correlation_AllDirection_offset7_SD']

['GreyLevelNonuniformity_angle90_offset1']

['GLCMEnergy_angle135_offset7']

['RunLengthNonuniformity_angle45_offset1']

['HighGreyLevelRunEmphasis_angle45_offset4']

['InverseDifferenceMoment_angle45_offset4']

['ClusterProminence_angle45_offset4']

['differenceEntropy']

['ClusterShade_AllDirection_offset7']

['HighGreyLevelRunEmphasis_AllDirection_offset4']

['HaralickCorrelation_angle45_offset4']

['GreyLevelNonuniformity_angle45_offset7']

['RunLengthNonuniformity_angle135_offset1']

['Inertia_AllDirection_offset1_SD']

['LowGreyLevelRunEmphasis_AllDirection_offset7_SD']

['HaralickCorrelation_angle0_offset4']

['Variance']

['FrequencySize']

['ShortRunLowGreyLevelEmphasis_angle0_offset7']

['GLCMEntropy_angle90_offset1']

['GreyLevelNonuniformity_angle90_offset7']

['InverseDifferenceMoment_AllDirection_offset4']

['GLCMEnergy_angle0_offset1']

['contrast']

['ShortRunLowGreyLevelEmphasis_angle45_offset1']

['LongRunHighGreyLevelEmphasis_AllDirection_offset7_SD']

['ShortRunEmphasis_angle135_offset4']

['HighGreyLevelRunEmphasis_angle45_offset1']

['histogramEntropy']

['Correlation_angle0_offset7']

['Correlation_AllDirection_offset1_SD']

['Inertia_angle0_offset4']

['LowGreyLevelRunEmphasis_angle90_offset1']

['LowGreyLevelRunEmphasis_angle135_offset1']

['LowGreyLevelRunEmphasis_angle45_offset4']

['GLCMEntropy_angle135_offset7']

['ShortRunEmphasis_AllDirection_offset7_SD']

['InverseDifferenceMoment_AllDirection_offset7']

['ShortRunEmphasis_angle0_offset7']

['sumVariance']

['LongRunHighGreyLevelEmphasis_angle0_offset7']

['InverseDifferenceMoment_angle45_offset1']

['LowGreyLevelRunEmphasis_AllDirection_offset7']

['VolumeCount']

['LowGreyLevelRunEmphasis_AllDirection_offset4']

['VoxelValueSum']

['ClusterProminence_angle135_offset1']

['GLCMEnergy_AllDirection_offset1']

['LongRunHighGreyLevelEmphasis_AllDirection_offset1_SD']

['HaraVariance']

['ShortRunLowGreyLevelEmphasis_angle90_offset1']

['LowGreyLevelRunEmphasis_angle90_offset7']

['GLCMEnergy_angle135_offset1']

['GLCMEnergy_angle0_offset7']

['GLCMEntropy_angle45_offset7']

['ClusterProminence_angle45_offset1']

['LowGreyLevelRunEmphasis_angle0_offset7']

['ShortRunLowGreyLevelEmphasis_angle90_offset4']

['inverseDifferenceMoment']

['InverseDifferenceMoment_angle135_offset1']

['ClusterShade_angle45_offset4']

['LowGreyLevelRunEmphasis_angle45_offset1']

['ClusterShade_AllDirection_offset4_SD']

['RunLengthNonuniformity_angle45_offset4']

['ShortRunEmphasis_angle90_offset4']

['Inertia_angle45_offset7']

['HighGreyLevelRunEmphasis_AllDirection_offset7']

['ClusterProminence_angle135_offset7']

['RunLengthNonuniformity_angle90_offset7']

['GreyLevelNonuniformity_angle0_offset1']

['ClusterShade_angle90_offset1']

['SphericalDisproportion']

['LongRunHighGreyLevelEmphasis_angle0_offset1']

['VolumeCC']

['ClusterShade_AllDirection_offset1']

['Inertia_angle135_offset4']

['HighGreyLevelRunEmphasis_angle90_offset1']

['Percentile95']

['ClusterProminence_AllDirection_offset1_SD']

['LongRunEmphasis_AllDirection_offset1']

['ClusterProminence_angle135_offset4']

['GreyLevelNonuniformity_angle135_offset1']

['RunLengthNonuniformity_angle0_offset4']

['sumEntropy']

['GLCMEntropy_AllDirection_offset1_SD']

['Inertia_angle0_offset7']

['Percentile85']

['HaralickCorrelation_angle135_offset1']

['HighGreyLevelRunEmphasis_angle90_offset4']

['GreyLevelNonuniformity_angle0_offset7']

['GLCMEntropy_AllDirection_offset4']

['ClusterProminence_angle0_offset1']

['GLCMEnergy_angle0_offset4']

['ShortRunLowGreyLevelEmphasis_angle135_offset7']

['LongRunHighGreyLevelEmphasis_angle135_offset1']

['InverseDifferenceMoment_AllDirection_offset1']

['RunLengthNonuniformity_angle0_offset1']

['ClusterProminence_AllDirection_offset4']

['GreyLevelNonuniformity_AllDirection_offset7_SD']

['RunLengthNonuniformity_angle90_offset1']

['GLCMEnergy_angle90_offset7']

['GreyLevelNonuniformity_AllDirection_offset4']

['ShortRunEmphasis_angle0_offset4']

['GLCMEnergy_angle45_offset4']

['ShortRunLowGreyLevelEmphasis_angle0_offset1']

['LongRunHighGreyLevelEmphasis_angle90_offset4']

['Correlation_AllDirection_offset1']

['InverseDifferenceMoment_angle135_offset7']

['Inertia_angle45_offset1']

['LongRunHighGreyLevelEmphasis_angle45_offset1']

['ClusterProminence_AllDirection_offset1']

['HaralickCorrelation_angle0_offset7']

['ClusterShade_angle135_offset7']

['LongRunEmphasis_AllDirection_offset4']

['ShortRunLowGreyLevelEmphasis_AllDirection_offset1']

['GLCMEntropy_angle90_offset7']

['LongRunHighGreyLevelEmphasis_AllDirection_offset4_SD']

['LongRunEmphasis_AllDirection_offset4_SD']

['LongRunEmphasis_angle90_offset1']

['ClusterShade_angle0_offset7']

['Correlation_angle45_offset1']

['Quantile0.975']

['ShortRunEmphasis_AllDirection_offset4_SD']

['HighGreyLevelRunEmphasis_angle0_offset1']

['ShortRunLowGreyLevelEmphasis_angle135_offset4']

['HaralickCorrelation_angle135_offset4']

['ShortRunEmphasis_AllDirection_offset4']

['GreyLevelNonuniformity_angle45_offset1']

['ShortRunEmphasis_angle45_offset7']

['HaralickCorrelation_AllDirection_offset1']

['GLCMEntropy_angle45_offset1']

['GLCMEnergy_angle45_offset7']

['HighGreyLevelRunEmphasis_angle45_offset7']

['Compactness1']

['GLCMEnergy_angle45_offset1']

['LowGreyLevelRunEmphasis_angle135_offset4']

['HaralickCorrelation_angle45_offset7']

['HaralickCorrelation_AllDirection_offset4']

['Correlation_angle135_offset1']

['LongRunLowGreyLevelEmphasis_AllDirection_offset1_SD']

['LongRunEmphasis_angle0_offset1']

['Inertia_angle0_offset1']

['LongRunEmphasis_AllDirection_offset1_SD']

['InverseDifferenceMoment_angle90_offset4']

['Inertia_angle135_offset1']

['stdDeviation']

['LongRunHighGreyLevelEmphasis_angle90_offset1']

['HighGreyLevelRunEmphasis_angle135_offset1']

['ClusterShade_angle135_offset4']

['RunLengthNonuniformity_angle90_offset4']

['ShortRunLowGreyLevelEmphasis_AllDirection_offset7']

['uniformity']

['ShortRunLowGreyLevelEmphasis_angle135_offset1']

['SurfaceVolumeRatio']

['GLCMEntropy_angle45_offset4']

['RunLengthNonuniformity_AllDirection_offset1_SD']

['RunLengthNonuniformity_AllDirection_offset7']

['ClusterShade_angle135_offset1']

['LongRunEmphasis_angle0_offset4']

['ClusterProminence_angle45_offset7']

['HaralickCorrelation_AllDirection_offset7']

['GLCMEnergy_angle90_offset1']

['ShortRunEmphasis_angle90_offset7']

['Inertia_AllDirection_offset4']

['InverseDifferenceMoment_angle90_offset1']

['RunLengthNonuniformity_AllDirection_offset4']

['ShortRunLowGreyLevelEmphasis_angle0_offset4']

['Range']

['GreyLevelNonuniformity_angle90_offset4']

['LongRunHighGreyLevelEmphasis_angle45_offset4']

['VolumeMM']

['GLCMEnergy_AllDirection_offset7']

['LowGreyLevelRunEmphasis_angle90_offset4']

['GLCMEntropy_AllDirection_offset1']

['GLCMEnergy_AllDirection_offset1_SD']

['InverseDifferenceMoment_angle0_offset4']

['AngularSecondMoment']

['InverseDifferenceMoment_angle45_offset7']

['LowGreyLevelRunEmphasis_angle45_offset7']

['LowGreyLevelRunEmphasis_angle0_offset1']

['RunLengthNonuniformity_angle135_offset4']

['ShortRunLowGreyLevelEmphasis_AllDirection_offset4']

['LongRunHighGreyLevelEmphasis_angle0_offset4']

['ShortRunEmphasis_AllDirection_offset7']

['HaraEntroy']

['ClusterProminence_angle0_offset4']

['ClusterProminence_AllDirection_offset7']

['SurfaceArea']

['GLCMEntropy_AllDirection_offset7']

['HaralickCorrelation_angle135_offset7']

['RunLengthNonuniformity_angle0_offset7']

['InverseDifferenceMoment_angle90_offset7']

['ClusterShade_AllDirection_offset7_SD']

['GreyLevelNonuniformity_AllDirection_offset4_SD']

['RunLengthNonuniformity_AllDirection_offset7_SD']

['RunLengthNonuniformity_angle45_offset7']

['RunLengthNonuniformity_AllDirection_offset4_SD']

['LongRunEmphasis_angle45_offset1']

['LongRunHighGreyLevelEmphasis_AllDirection_offset4']

['ShortRunLowGreyLevelEmphasis_angle90_offset7']

['ShortRunEmphasis_angle90_offset1']

['ClusterProminence_angle90_offset4']

['HighGreyLevelRunEmphasis_angle0_offset4']

['ClusterProminence_angle90_offset1']

['GLCMEntropy_angle135_offset1']

['ShortRunLowGreyLevelEmphasis_angle45_offset4']

['histogramEnergy']

['LowGreyLevelRunEmphasis_AllDirection_offset1']

['LowGreyLevelRunEmphasis_angle0_offset4']

['HaralickCorrelation_angle0_offset1']

['GLCMEntropy_angle0_offset4']

['ClusterProminence_AllDirection_offset4_SD']

['LongRunHighGreyLevelEmphasis_AllDirection_offset1']

['ShortRunLowGreyLevelEmphasis_AllDirection_offset7_SD']

['GLCMEnergy_angle135_offset4']

['HaralickCorrelation_AllDirection_offset1_SD']

['ClusterShade_angle0_offset1']

['Inertia_AllDirection_offset7']

['InverseDifferenceMoment_angle0_offset7']

['GreyLevelNonuniformity_AllDirection_offset1_SD']

['ShortRunHighGreyLevelEmphasis_AllDirection_offset7_SD']

['ShortRunEmphasis_angle45_offset4']

['Inertia_angle90_offset1']

['ShortRunEmphasis_angle0_offset1']

['Correlation_angle0_offset1']

['sumAverage']

['RunLengthNonuniformity_angle135_offset7']

['ClusterShade_angle0_offset4']

['GLCMEnergy_AllDirection_offset7_SD']

['differenceVariance']

['HighGreyLevelRunEmphasis_angle135_offset7']

['GreyLevelNonuniformity_AllDirection_offset1']

['ShortRunHighGreyLevelEmphasis_AllDirection_offset4_SD']

['HighGreyLevelRunEmphasis_AllDirection_offset4_SD']

['GLCMEntropy_angle90_offset4']

['Inertia_angle45_offset4']

['GreyLevelNonuniformity_angle45_offset4']

['RunLengthNonuniformity_AllDirection_offset1']

['HighGreyLevelRunEmphasis_angle0_offset7']

['Inertia_angle90_offset4']

['HighGreyLevelRunEmphasis_AllDirection_offset1']

['Inertia_AllDirection_offset4_SD']

['Correlation_AllDirection_offset4_SD']

['GLCMEnergy_AllDirection_offset4']

['ClusterShade_angle45_offset1']

['HaralickCorrelation_angle90_offset4']

['ShortRunLowGreyLevelEmphasis_AllDirection_offset4_SD']

['ShortRunEmphasis_angle135_offset1']

['HighGreyLevelRunEmphasis_angle90_offset7']

['LowGreyLevelRunEmphasis_angle135_offset7']

['Compactness2']

['Correlation_AllDirection_offset7']

['GLCMEnergy_angle90_offset4']

['ClusterShade_angle90_offset7']

['GreyLevelNonuniformity_angle135_offset4']

['Percentile90']

['MaxIntensity']

['ClusterProminence_angle90_offset7']

['InverseDifferenceMoment_angle0_offset1']

['ClusterShade_angle45_offset7']

['GLCMEntropy_angle135_offset4']]

Then the least absolute shrinkage and selection operator (LASSO) was applied to select

the most useful features from the primary data.

parameters setted: {'alpha': 0.008957598878169343}

num of remained features: 7

remained features:

['Sphericity']

['Inertia_angle135_offset7']

['histogramEntropy']

['Correlation_angle0_offset7']

['GLCMEntropy_angle135_offset7']

['VoxelValueSum']

['MaxIntensity']
